# Evolutionary Convergence on Highly-Conserved 3′ Intron Structures in Intron-Poor Eukaryotes and Insights into the Ancestral Eukaryotic Genome

**DOI:** 10.1371/journal.pgen.1000148

**Published:** 2008-08-08

**Authors:** Manuel Irimia, Scott William Roy

**Affiliations:** 1 Departament de Genetica, Facultat de Biologia, Universitat de Barcelona, Barcelona, Spain; 2 National Center for Biotechnology Information, National Library of Medicine, National Institutes of Health, Bethesda, Maryland, United States of America; Fred Hutchinson Cancer Research Center, United States of America

## Abstract

The presence of spliceosomal introns in eukaryotes raises a range of questions about genomic evolution. Along with the fundamental mysteries of introns' initial proliferation and persistence, the evolutionary forces acting on intron sequences remain largely mysterious. Intron number varies across species from a few introns per genome to several introns per gene, and the elements of intron sequences directly implicated in splicing vary from degenerate to strict consensus motifs. We report a 50-species comparative genomic study of intron sequences across most eukaryotic groups. We find two broad and striking patterns. First, we find that some highly intron-poor lineages have undergone evolutionary convergence to strong 3′ consensus intron structures. This finding holds for both branch point sequence and distance between the branch point and the 3′ splice site. Interestingly, this difference appears to exist within the genomes of green alga of the genus *Ostreococcus*, which exhibit highly constrained intron sequences through most of the intron-poor genome, but not in one much more intron-dense genomic region. Second, we find evidence that ancestral genomes contained highly variable branch point sequences, similar to more complex modern intron-rich eukaryotic lineages. In addition, ancestral structures are likely to have included polyT tails similar to those in metazoans and plants, which we found in a variety of protist lineages. Intriguingly, intron structure evolution appears to be quite different across lineages experiencing different types of genome reduction: whereas lineages with very few introns tend towards highly regular intronic sequences, lineages with very short introns tend towards highly degenerate sequences. Together, these results attest to the complex nature of ancestral eukaryotic splicing, the qualitatively different evolutionary forces acting on intron structures across modern lineages, and the impressive evolutionary malleability of eukaryotic gene structures.

## Author Summary

The spliceosomal introns that interrupt eukaryotic genes show great number and sequence variation across species, from the rare, highly uniform yeast introns to the ubiquitous and highly variable vertebrate intron sequences. The causes of these differences remain mysterious. We studied sequences of intron branch points and 3′ termini in 50 eukaryotic species. All intron-rich species exhibit variable 3′ sequences. However, intron-poor species range from variable sequences, to uniform branch point motifs, to uniform branch point motifs in uniform positions along the intronic sequence. This is a more complex pattern than the clear relationship between intron number and 5′ intron sequence uniformity found previously. The correspondence of sequence uniformity and intron number extends to species of the green algal genus *Ostreococcus*, in which the single intron-rich genomic region shows far more variable intron sequences than in the otherwise intron-poor genome. We suggest that different concentrations of spliceosomal complexes may explain these differences. In addition, we report the existence of 3′ polyT tails in diverse eukaryotic protists, suggesting that this structure is ancestral. Together, these results underscore the complexity of ancestral eukaryotic splicing, the qualitatively different evolutionary forces acting on intron sequences in modern eukaryotes, and the impressive evolutionary malleability of eukaryotic genes.

## Introduction

Spliceosomal introns are genomically-encoded sequences that are removed from RNA transcripts by the spliceosome, a massive RNA-protein complex. The spliceosome and spliceosomal introns are common and ancestral to eukaryotes [1]–[4], however spliceosomal organization shows striking divergence across species. Intron number per genome differs by orders of magnitude, from fewer than ten known introns in the genomes of some protist species [4],[5] to nearly ten per gene in some metazoans [6],[7]. Introns also vary dramatically in length, from the ‘bonzaied’ 19 nt introns of the *Bigelowiella natans* nucleomorph [8] to the giant kilobases-long introns of humans and other mammals.

**Figure 1:**
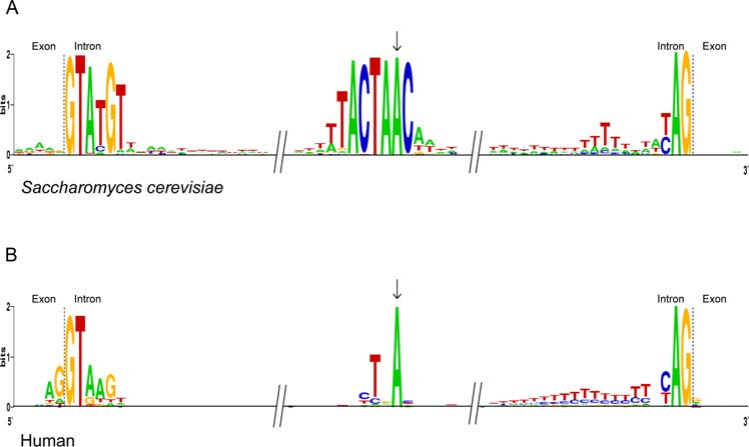
Intron sequence structures. Examples of a species with (A) strong consensus structures (*S. cerevisiae*), and (B) weakly conserved structures (human).

Intron sequence elements, which are important for intron recognition by the RNA and protein components of the spliceosome, also vary significantly across species. In some species, the consensus of an intron sequence is largely restricted to an initial 5′ GT dinucleotide (the “donor” site), a terminal 3′ AG (the “acceptor”), and a degenerate few nt “branch point” site, lying somewhere within the intron. In other species, sequences are more conserved. For instance, in the apicomplexan parasite *Cryptosporidium*, 84% of introns begin with the most common sixmer GTAAGT, enabling close complementary base pairing with the U1 RNA of the spliceosome (compared with only 14% for humans; see examples in Figure 1). We previously studied the phylogenetic pattern of conservation of 5′ intronic sequences and found a strong correspondence between species with very few introns and those with such strong 5′ consensus sequences [9].

**Figure 2:**
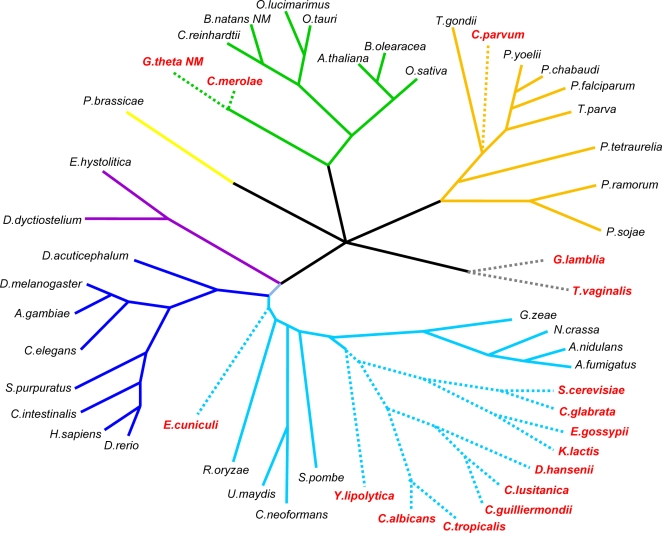
Phylogenetic relationship between the species included in this study. Consensus phylogenetic tree of the species included in this study. Red species names and discontinuous branches indicate intron-poor species. Note that representatives of all eukaryotic supergroups have been included in this study. Based on [47],[48].

Clear differences are also observed in other structures. For instance, branch points vary across species from a highly conserved seven-mer (TACTAAC) in hemiascomycetous yeasts (e.g., *Saccharomyces cerevisiae*) to several lineages in which previous studies have failed to find branch points [10],[11]. Here we report studies of the evolution of 3′ intron structures including the branch point, poly-pyrimidine tract and 3′ splice site across 50 species spanning all major eukaryotic kingdoms (opisthokonts, amoebozoans, red and green plants, chromalveolates, excavates and rhizarians, Figure 2).

## Results

### Branch Point Consensus Strength in Intron-Poor Species

The branch point is an internal intronic sequence that initiates the splicing event through a hydrophilic attack by an adenosine 2′ hydroxyl group at the 5′ splice site [12],[13]. As with donor sites, branch point consensus strength varies across species: 84% of *Saccharomyces cerevisiae* introns utilize the sequence TACTAAC and 94% have ACTAAC, compared to fewer than 20% of human introns with the exact same sixmer at the branch point site (Figure 1), and with *Caenorhabditis* nematodes, where no branch points have been identified.

We first studied intron branch points in available genomes from intron-poor species (Table 1; defined, as before, as those species with ∼0.1 or fewer introns per gene on average [9]). Many of these species exhibited clear branch point consensus sequences. First, we found that the *S. cerevisiae* branch point consensus ACTAAC (throughout, the putative branch point A is underlined) is found in all fully-sequenced hemiascomycetous yeasts, with 66–100% of introns in each species containing this motif. Extended branch points of up to eight bases were found in some species (Table 1). We also found an extended single ACTAACC branch point motif in all 26 known introns of the red alga *Cyanidioschyzon merolae*. Other intron-poor species containing strong putative branch point motifs were the two excavate species: 32/34 (94.12%) probable introns (see Materials and Methods) in the flagellated protist *Trichomonas vaginalis* use WCTAAC, and all five known introns in *Giardia lamblia* (four published plus one unpublished instance) contain CTRACA. Finally, excluding two questionable predicted introns (see Discussion), all 13 introns in the microsporidium parasite *Encephalitozoon cuniculi* contain a TAAYTT hexamer (9/13 have CTAAYTT). Thus all lineages with strong branch points conformed to a general WCTRAYN consensus.

**Table 1:** Putative branch point consensus in the 16 studied intron-poor species.

Hemiacsomycete yeasts	
*Candida albicans*	TACTAAC
*Candida glabrata*	TACTAACA
*Candida tropicalis*	ACTAAC
*Candida guilliermondii*	ACTAAC
*Candida lusitaniae*	ACTAAC
*Debaryomyces hansenii*	TACTAAC
*Eremothecium gossypii*	ACTAAC
*Kluyveromyces lactis*	TACTAAC
*Saccharomyces cerevisiae*	TACTAAC
*Yarrowia lipolytica*	ACTAAC
Red algae	
*Cyanidioschyzon merolae*	ACTAACC
*Guillardia theta NM*	Not clear
Excavates	
*Trichomonas vaginalis*	WCTAACA
*Giardia lamblia*	CTRACA
Microsporidians	
*Encephalitozoon cuniculi*	CTAAYTT
Apicomplexans	
*Cryptosporidium parvum*	Not clear
CONSENSUS	WCTRAYN

The active adenosine is underlined.

However, in at least one intron-poor lineage we failed to find such clear branch point sequences. Visual inspection and computational analysis (see below) of the intron-poor apicomplexan parasite *Cryptosporidium parvum* failed to reveal a potential branch point site. The case for the few introns of the *Guillardia theta* nucleomorph is less clear. 14/16 predicted *G. theta* introns contain a YAAY branch point-like sequence between 2 and 6 nucleotides from the acceptor AG (compared to only 2/16 that contain such a motif within the next 10 positions (7 to 17)). Intriguingly, a second YAAY signal exists further upstream – 8/16 introns have a YAAY 24–28 nt from the 3′ terminus. The single known intron in each of the sequenced Trypanosomatid genomes does not show a clear branch point structure; however it is of note that despite having few *cis*-spliced introns, these species do have large numbers of 3′ splicing boundaries due to the ubiquity of spliceosome-mediated spliced leader trans-splicing [14],[15].

### Branch Point Consensus Strength in Intron-Rich Species

We next studied conservation of branch points in 33 more intron-rich species. We studied the occurrence of motifs conforming to the consensus branch point WCTRAY or CTRAYN with varying levels of two-fold degeneracy (i.e. allowing two possible nucleotides at each site). For instance, the ACTAAC hexamer common to all *C. merolae* introns has no degeneracy, whereas the CTRACA motif of *G. lamblia* has one degenerate site, and the general consensus WCTRAY contains three two-fold degenerate sites. As with the other sites, either one or two nucleotides at a time were allowed at the final “N” site (CTRAYA, CTRAYC, CTRAYR, CTRAYY…).

For each species, and for each level of degeneracy (zero to three degenerate sites), we calculated the fraction of introns that contain the same motif within the last 200 nt of the intron (see Methods). Then, for each level of degeneracy, we identified the motif that was present in the largest fraction of introns for that species. These values are given in Table 2. As expected, the intron-poor species discussed above give high values at all levels of degeneracy. All intron-poor species with strong consensus discussed above have values of at least 72% for one-site degenerate branch point motifs.

**Table 2:** Intron features for the 50 eukaryotic species included in this study.

SPECIES NAME	% of introns containing most common branch point motif for given number of degenerate sites				Introns studied	%AT	INTRON LENGTH		
	0	1	2	3			Median	Aver	Std Dev
*Aspergillus fumigatus*	10.97% (actgac)	17.96% (ctgacw)	29.99% (ctracw)	43.18% (ctrayw)	18267	54.08	61	100.06	297.62
*Anopheles gambiae*	6.12% (actaat)	11.12% (wctaat)	18.04% (wctaay)	24.58% (wctray)	33443	56.18	91	1044.3	3533.97
*Aspergillus nidulans*	8.33% (ctaaca)	15.79% (actrac)	28.32% (ctracw)	42.78% (ctrayw)	25500	54.22	63	101.52	99.94
*Arabidopsis thaliana*	8.23% (tctgat)	14.95% (tctrat)	23.36% (wctrat)	30.99% (wctray)	134535	68.04	98	157.66	163.99
*Bigellowiella natans NM*	1.16% (ctaata)	1.39% (ctaatm)	1.39% (wctaay)	1.39% (wctray)	860	87.96	19	19.1	0.57
*Cyanidioschyzon merolae*	100.00% (actaac)	100.00% (wctaac)	100.00% (wctrac)	100.00% (wctray)	27	46.82	234	247.89	229.06
*Candida albicans*	95.43% (actaac)	96.45% (actaay)	96.95% (wctaay)	96.95% (wctray)	197	70.61	100	196.28	174.31
*Caenorhabditis elegans*	7.09% (ctaatt)	10.43% (ctratt)	15.13% (ctratw)	20.34% (ctrayw)	102420	68.07	65	303.49	677.7
*Candida glabrata*	66.25% (actaac)	72.50% (actaay)	75.00% (wctaay)	78.75% (wctray)	80	67.48	467	462.65	295.64
*Chlamydomonas reinhardtii*	7.29% (ctgacc)	13.22% (ctgacs)	19.51% (ctgays)	25.64% (ctrays)	105401	36.14	238	371.60	527.54
*Ciona intestinalis*	8.37% (ctaatt)	14.97% (ctaatw)	23.47% (ctaayw)	32.71% (ctrayw)	48942	66.74	337	540.56	1117.91
*Cryptococcus neoformans*	10.08% (ctgaca)	17.12% (ctgacw)	27.49% (ctgayw)	39.66% (ctrayr)	33059	56.76	55	67.36	55.1
*Cryptosporidium parvum*	9.39% (ctaatt)	14.08% (ctaatw)	17.37% (ctaayw)	20.19% (wctray)	213	77.96	61	82.08	72.97
*Dictyostelium discoideum*	18.66% (actaat)	24.67% (wctaat)	31.89% (wctaay)	33.31% (wctray)	17300	89.13	103	142.85	158.36
*Debaryomyces hansenii*	71.83% (actaac)	77.18% (actaay)	81.41% (wctaay)	83.38% (wctray)	346	66.23	89	171.86	166.52
*Drosophila melanogaster*	8.73% (actaat)	14.68% (wctaat)	21.63% (wctaay)	28.71% (wctray)	41049	59.93	71	892.93	3802.06
*Danio rerio*	8.57% (ctgatt)	15.17% (ctratt)	26.26% (wctrat)	37.70% (ctrayw)	200465	64.47	984	2864.33	11125.47
*Encephalitozoon cuniculi*	38.46% (ctaatt)	69.23% (ctaayt)	69.23% (ctrayt)	69.23% (ctrayw)	13	60.77	32	35.77	8.48
*Eremothecium gossypii*	82.03% (actaac)	85.71% (actrac)	87.10% (wctrac)	89.40% (wctray)	228	46.56	63	113.3	96.77
*Entamoeba histolytica*	5.95% (actaat)	9.09% (wctaat)	12.16% (wctaay)	14.95% (wctray)	3053	79.48	58	97.5	179
*Giardia lamblia*	60.00% (ctaaca)	100.00% (ctraca)	100.00% (ctraya)	100.00% (ctraym)	5	50.8	32	85	70.35
*Guillardia theta NM*	6.06% (actaac)	6.06% (wctaac)	6.06% (wctrac)	6.06% (wctray)	17	84.8	46	46.27	2.67
*Gibberella zeae*	15.88% (ctaaca)	24.62% (actrac)	38.10% (ctracw)	50.15% (ctrayw)	25853	56.92	56	92.84	94.09
*Homo sapiens*	7.90% (ctgacc)	14.06% (ctgacy)	22.80% (ctgayy)	34.32% (ctrayw)	197835	58.5	1507	5429.85	19221.6
*Kluyveromyces lactis*	99.22% (actaac)	100.00% (wctaac)	100.00% (wctrac)	100.00% (wctray)	130	65.53	266	344.69	252.34
*Neurospora crassa*	15.38% (ctaaca)	26.83% (ctraca)	42.59% (ctracm)	50.83% (ctraym)	5991	52.11	83	130.57	122.29
*Ostreococcus lucimarinus*	41.15% (actgac)	42.97% (actrac)	44.93% (wctrac)	47.16% (wctray)	2148	37.24	111	168.4	274.23
*Ostreococcus tauri*	9.55% (actgac)	10.53% (actrac)	11.83% (wctgay)	14.02% (wctray)	6450	39.27	52	83.39	85.39
*Oryza sativa*	7.86% (ctaatt)	14.40% (ctratt)	23.80% (wctrat)	33.73% (ctrayw)	102728	61.56	171	406.55	594.32
*Paramecium tetraurelia*	1.66% (tctaat)	2.72% (ctaatw)	3.12% (ctaayw)	3.55% (ctrayw)	90253	83.77	25	25.14	3.06
*Plasmodium chabaudi*	5.46% (ctaatt)	10.18% (ctaatw)	12.73% (ctratw)	16.02% (ctrayw)	6738	77.89	121	152.82	135.06
*Plasmodium falciparum*	3.30% (ctaatt)	5.93% (ctaatw)	7.31% (ctratw)	8.50% (ctrayw)	7370	87.77	144	176.73	136.85
*Phytophthora ramorum*	5.94% (ctgacg)	10.54% (ctgack)	19.15% (ctrack)	30.72% (ctrayk)	12829	49.8	74	132.37	371.64
*Phytophthora sojae*	5.42% (ctgacg)	9.87% (ctgack)	17.69% (ctrack)	26.92% (ctrayk)	17360	48.09	78	131.22	444.38
*Plasmodium yoelii*	5.37% (ctaata)	10.09% (ctaatw)	12.77% (ctratw)	15.76% (ctrayw)	7890	79.73	133	207.25	277.27
*Rhizopus oryzae*	4.77% (actaat)	8.78% (wctaat)	13.62% (wctrat)	20.37% (wctray)	40358	71.06	57	79.08	56.89
*Saccharomyces cerevisiae*	94.40% (actaac)	95.52% (actaay)	96.64% (actray)	97.39% (wctray)	260	66.46	148	241.53	175.93
*Schizosaccharomyces pombe*	21.61% (actaac)	35.26% (actaay)	50.46% (wctaay)	56.22% (wctray)	4722	71.47	56	82.27	68.21
*Strongylocentrotus purpuratus*	8.52% (tctaat)	16.08% (tctrat)	26.67% (wctrat)	36.30% (wctray)	120111	64.37	748	1628.48	4405.72
*Toxoplasma gondii*	4.54% (tctgac)	8.45% (tctgay)	14.06% (ctgayk)	17.75% (ctrayk)	27085	50.8	490	578.26	468.81
*Theileria parva*	9.03% (ctaatt)	14.38% (ctaatw)	18.13% (ctaayw)	21.43% (ctrayw)	3390	76.05	63	91.42	102.53
*Trichomonas vaginalis*	94.12% (ctaaca)	97.06% (ctaacm)	97.06% (ctracm)	97.06% (ctraym)	34	71.94	104	101.27	27.13
*Ustilago maydis*	11.64% (actgac)	17.78% (ctgacm)	24.77% (ctgaym)	29.50% (ctraym)	4859	49.67	95	126.88	109.79
*Yarrowia lipolytica*	70.72% (actaac)	78.73% (wctaac)	80.25% (wctrac)	82.87% (wctray)	719	51.48	204	268.75	225.59
*Brassica oleracea*	7.67% (tctgat)	12.88% (tctrat)	20.27% (wctrat)	27.12% (wctray)	1096*	67.97	93	165.67	316.9
*Candida tropicalis*	100.00% (actaac)	100.00% (wctaac)	100.00% (wctrac)	100.00% (wctray)	34*	72.19	81	199.26	175.43
*Candida guilliermondii*	76.92% (actaac)	84.62% (actaay)	84.62% (wctaay)	84.62% (wctray)	13*	63.51	65	100.54	94.49
*Candida lusitaniae*	90.00% (actaac)	90.00% (wctaac)	90.00% (wctrac)	90.00% (wctray)	10*	63.17	98	110.7	58.67
*Dicyema acuticephalum*	4.88% (ctaatt)	7.32% (ctwatt)	9.76% (ctwayt)	12.20% (ctwayk)	41*	69.89	25	24.93	2.51
*Plasmodiophora brassicae*	8.14% (tctgac)	13.95% (ctgacr)	22.09% (ctgayk)	26.74% (ctrayk)	86*	49.87	55	55.66	7.01
									

*: No full genome sequence available.

**Figure 3:**
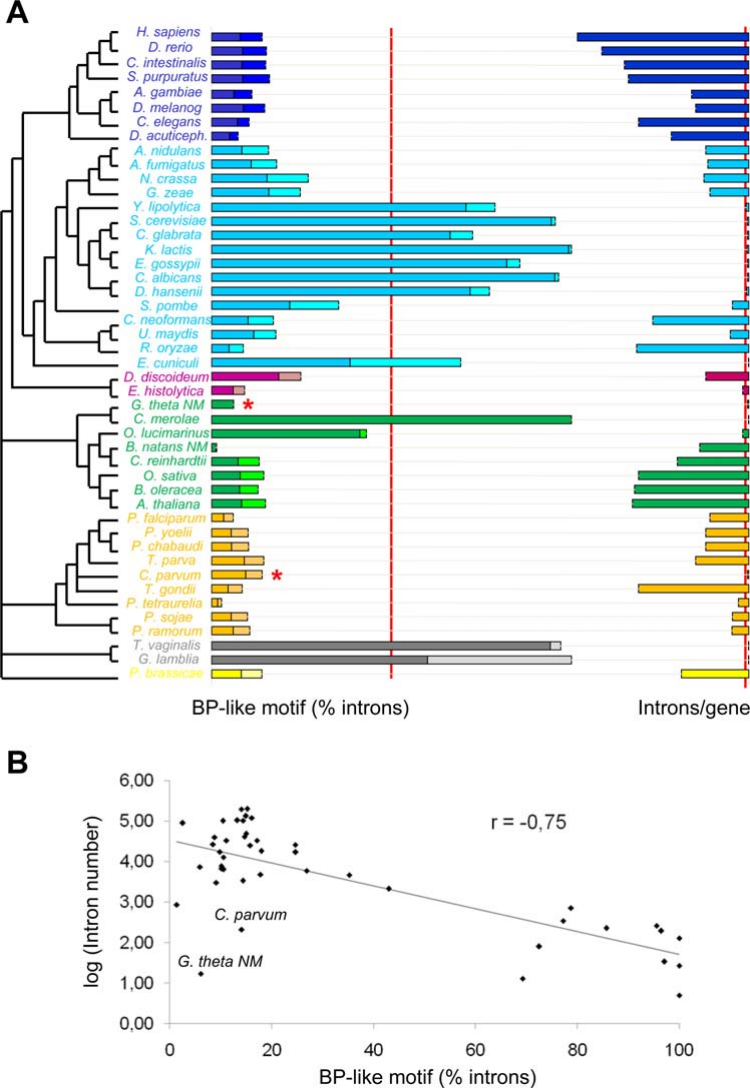
Relation between branch point site consensus strength and intron number. (A) Percentage of introns with the most common BP-like hexamer across eukaryotes. Dark colored bars show the percentage of introns with the most common branch-point hexamer; light colored bars show the additional introns allowing for two-fold degeneracy at one of the six sites. The left red bar separates species with ‘strong’ BP motifs (>50% of introns) from species with weaker BP motifs. The right red bar separate extremely intron-poor species (< 0.15 introns per gene). Asterisks (*) mark the two exception to the correspondence between the two variables (strong BP/intron-poor vs. weak BP/intron-rich). (B) Logarithm of total intron number (Y-axis) and percentage of introns showing the most common potential branch point motif with one site two-fold degeneracy (X-axis) are plotted for all fully sequenced studied genomes. The species cluster in two different groups (with two exceptions) - intron-rich/weak BP consensus, intron-poor/strong BP consensus. The two intron-poor species not showing strong consensus branch points, *G. thetha* NM and *C. parvum*, are labeled. The line corresponds to a lineal regression analysis, showing the negative correlation (r = −0.75) between intron numbers and branch point consensus conservation.

By contrast, every studied intron-rich species shows much lower scores, with lower than 22% of introns with the same putative branch point motif (for example, 8.73% (ACTAAT) in *Drosophila melanogaster* or 10.97% (ACTGAC) in *Aspergillus fumigatus*), and less than 36% allowing one degenerate site. This relation between intron numbers and branch point consensus strength is underscored in Figure 3. The species are clearly distributed in two main groups (with two exceptions, *G. thetha* NM and *C. parvum* (red asterisks)): intron-rich/weak branch point consensus and intron-poor/strong branch consensus (intron-poor, fewer than 0.15 introns per gene; strong branch points, same BP-like hexamer in more than 50% of introns (red lines in Figure 3A). Overall, there is a negative correlation (r = −0.75) between intron numbers and branch point consensus conservation (by a linear regression analysis, Figure 3B).

### The Peculiar Case of *Ostreococcus*

Only the species *Ostreococcus lucimarinus* appeared to represent an intermediate between strong and weak branch point species, with 41.15% of predicted introns containing the same branch point-like ACTGAC sequence (Table 2). However, given the high evolutionary distance of *O. lucimarinus* from other species with fully-sequenced genomes and the relatively small number of available transcript sequences, annotation in *O. lucimarinus* and distant congenitor *O. tauri* is difficult and some intron predictions may thus be unreliable. To identify a confident set of introns, we performed BLASTN searches of the predicted intron-containing coding sequences against the 17,592 available *O. lucimarinus* EST sequences. Further computational and manual filtering for ambiguities and potential problems associated with reverse transcriptase [16],[17] yielded a total of 560 confirmed intron sequences.

The confirmed introns show a stronger signal, with 50.7% containing the branch point sequence ACTGAC (and 37.9% showing an extended branch point GACTGACG). The pattern becomes even more striking when intron sequences are divided along the lines of the previously reported genomic heterogeneity of *O. lucimarinus*, with roughly half of chromosome 2 differing from the rest of the genome in a variety of ways including much higher intron density [18]. Confirmed chromosome 2 introns show very weak branch point signal, with only 4.6% sharing the same sixmer (CTGACG), while 87.2% of introns outside of chromosome 2 contain ACTGAC (Table 3), and 66.5% contain an extended GACTGACG motif. Confirmed introns outside of chromosome 2 also show very strong 5′ splice sites consistent with intron-poor structures (4.8 bits), whereas confirmed chromosome 2 introns show much weaker boundaries (1.9 bits) (Table 3). Notably, 5′ splice boundaries outside chromosome 2 exhibit the atypical consensus GTGCGTG, whereas chromosome 2 introns prefer a more typical GTRNGT.

**Table 3:** Intron signal features for different intron subsets of *O. lucimarinus.*

	Intron subset		Introns studied	5′ss strength (bits)	% of introns containing most common branch point motif for given number of degenerate sites			
					0	1	2	3
*O. lucimarinus*	All introns	All Chrom	2148	1.6	41.15% (actgac)	42.97% (actrac)	44.93% (wctrac)	47.16% (wctray)
		Chrom 2	1748	1.8	5.53% (ctgacg)	8.79% (ctgack)	15.08% (wctrac)	20.35% (wctray)
		Non-Chrom 2	396	1.8	49.12% (actgac)	50.65% (actrac)	51.56% (wctrac)	53.09% (wctray)
	EST confirmed	All Chrom	560	3.0	50.71% (actgac)	53.21% (actrac)	57.32% (wctrac)	60.36% (wctray)
		Chrom 2	241	1.9	4.56% (ctgacg)	8.30% (ctgack)	12.45% (ctrack)	17.01% (wctray)
		Non-Chrom 2	319	4.8	87.15% (actgac)	89.97% (actrac)	91.54% (wctrac)	93.10% (wctray)

In contrast to *O. lucimarinus*, predicted introns in *O. tauri* show far lower conservation of branch points (Table 2). The lack of EST sequences for this species makes confirmation difficult, however a few putative intron sequences identified by TBLASTN searches of intron-containing non-chromosome 2 *O. lucimarinus* genes against the *O. tauri* genome exhibited sequences similar to *O. lucimarinus* introns, suggesting that *O. tauri* may exhibit a similar pattern. Given this seeming discrepancy between all predicted and confirmed *O. tauri* introns, we excluded *O. tauri* from the analysis.

### Acceptor Site Conservation

**Figure 4:**
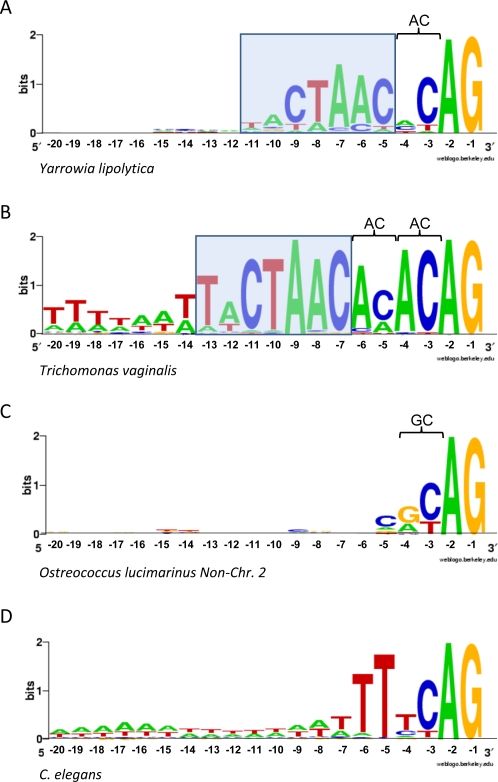
Highly constraint BP-AG distances in *Y. lipolytica* and *T. vaginalis*. (A) 3′ intron boundary in *Yaworria lipolytica*. (B) 3′ intron boundary in *Trichomonas vaginalis*. Note that the difference between both consensus is the existance of 1 (*Y. lipolytica*) or 2 (*T. vaginalis*) AC dinucleotides between the BP motif and the terminal AG. Consensus sequence for *G. lamblia* is similar to *T. vaginalis*. (C) 3′ intron boundary for confirmed introns from non-chromosome 2 genes in *O. lucimarinus*. (D) 3′ intron boundary in *C. elegans*.

Next, we studied acceptor sequence conservation within genomes. The 3′ sequences of spliceosomal introns generally show more similarity across species, with most species showing a terminal YAG, sometimes preceded by a poly-pyrimidine tract (Figure 1), although most fungal species and some others lack the poly-pyrimidine tract [19]. A survey of 50 widely diverged eukaryotic species showed 5 clear exceptions to this pattern. Three species, the protists *T. vaginalis* and *G. lamblia*, and *Yarrowia lipolytica*, a hemiascomycetes fungus (relatives of the baker's yeast *S. cerevisiae*), show strikingly similar patterns (Figure 4). The three species lack the 3′ polypyrimidine tract, and show clear anchoring of the branch point site at a specific number of basepairs away from the 3′ terminal AG (branchpoint-AG (BP-AG)) distance; 2 nt (AC) in *Y. lipolytica*, 4 nt (ACAC) in *T. vaginalis* and *G. lamblia*; Figure 4).

Non-chromosome 2 *O. lucimarinus* introns show a preference for CGCAG, though notably weaker than that found in the in the other three species (Figure 4). Interestingly, *O. lucimarinus* introns' conserved 3′ boundaries are associated with conserved BP-AG distance, as branch points for confirmed non-chromosome 2 *O. lucimarinus* introns show a broad peak ranging from around 20–35 bp (data not shown). It is interesting then that both *O. lucimarinus* and the constrained BP-AG distance species prefer a C at position –5 and a R at position –4. We were unable to find an explanation involving snRNA sequences for this preference. Finally, *C. elegans* introns also show stronger 3′ consensus, matching TTTCAG with nucleotides -6 and -5 significantly more conserved (Figure 4), as had been previously shown [20].

### 3′ Evolution in Hemiascomycetous Yeasts

**Figure 5:**
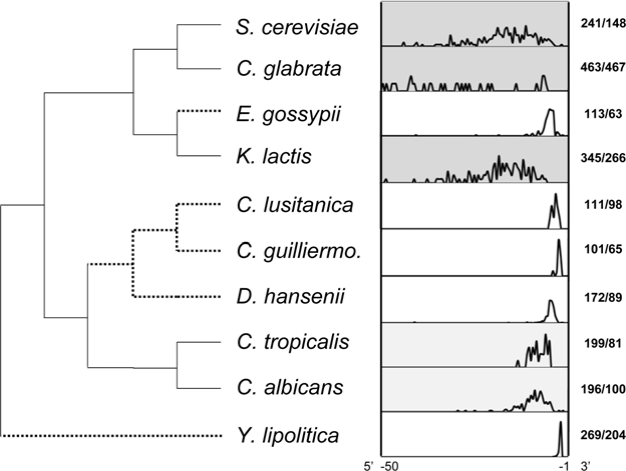
Phylogenetic distribution of BP-AG distance across hemiascomicetous yeasts. For each species, the distribution represents the fraction of introns with a certain distance to the AG from base -50 (most left) to base -1 (most right, terminal G). Grey background highlights relative lack of constraint BP-AG distance, and broken lines show the lineages that likely experienced the evolution of BP-AG distance constraint.

To better understand this pattern, we next studied available relatives of *Y. lipolytica*. We studied all six hemiascomycetes species with full genome annotations, as well as three additional *Candida* species for which some intron sequences were available. The species show pronounced differences, with three (including *S. cerevisiae*) showing large variations in BP-AG length and five species showing clear BP-AG constraint (differences in BP-AG length across hemiascomycetes was previously reported in [10]). In *Debaryomyces hansenii*, 65% of introns show a BP-AG distance between 6 and 8, and 88% of introns have a BP-AG distance between 5 and 10 nt. 75% of introns in *Eremothecium gossypii* with well defined branch points have BP-AG between 6 and 9 nt, with 66% between 6 and 11. The small number of available introns in *C. lusitaniae* and *C. guilliermondii* suggested preferred BP-AG distances of 4–5 and 3 nt, respectively. This BP-AG constraint could partially reflect differences in intron lengths, as mean/median lengths are lower for some of these species across the clade (Figure 5). However, the species with the clearest pattern of constraint, *Y. lipolytica,* has rather long introns relative to the other species.

Intriguingly, in *E. gossypii* introns, sequences between the BP and intron terminus varied considerably based on the BP-AG distance. The 30 introns with a BP-AG distance of 6 nt (the shortest distance with more than a few introns) showed a strong sequence consensus at the 3′ end of the sequence, with 80.0% having a G at position -4 (compared to only 39.3% for other introns; *p* = 0.00004 by a Fisher's Exact test), and 50.0% having an A at position -5 (compared to only 22.0% of other introns; *p* = 0.002 by a Fisher's Exact test). However no clear general trend towards stronger boundaries for short BP-AG introns was observed: introns with a BP-AG distance of 7 nt did now show a stronger 3′ consensus than introns with larger BP-AG distances. *Y. lipolytica* did not show differences in strength of 3′ sequence consensus for different BP-AG distances.

Mapping of BP-AG distances across hemiascomyces shows a complex phylogenetic pattern (Figure 5). The five species with strong BP-AG constraint are intermingled on the tree with the three less constrained lineages, suggesting convergent evolution of BP-AG constraint. Importantly, the “preferred” BP-AG distance varies across species – for *Y. lipolytica*, the most common BP-AG distance is 2 nt, compared to 3 in *C. guilliermondii*, 4–5 for *C. lusitaniae,* 6–8 for *E. gossypii* and 7–8 for *D. hanseii*.

It seems unlikely that a species with a strong preference for a certain BP-AG distance would convert to a different distance, since this would require indels of a very specific length occurring in dozens of already constrained introns. It seems more likely that this condition reflects ancestrally relatively unconstrained BP-AG distance, and convergent evolution of constrained BP-AG distances in different lineages.

### Introns in Intron-Rich Species Orthologous to Retained Introns in Intron-Poor Species

Convergent evolution of retained intron sequences in intron-poor species is likely due to a combination of two factors: preferential retention of introns with consensus-like (i.e. strong) sequences and change of retained intron sequences to consensus boundaries. However, the relative impact of these two factors is unknown. We attempted to address this issue by identifying introns in intron-rich species that were present at the exact homologous position to introns in any available intron-poor species, and thus are likely to be ancestral to both species. If strong consensus sequences in intron-poor species are due to differential retention of introns with conserved ancestral sequences, it is possible that orthologous introns in intron-rich species could retain some of this signal. For each intron-rich species (the apicomplexan *Toxoplasma gondii*, *H. sapiens*, *S. pombe*, *A. fumigatus*, and *A. thaliana*), we compared 5′ splice site strength and branch point conservation between introns putatively orthologous to introns retained in at least one intron-poor species and the total set of introns in these species. Significant differences for both intron structures were found for *A. fumigatus* and *T. gondii* introns (Table 4).

**Table 4:** Intron signal features.

	Intron subset		Introns studied	5′ss strength (bits)	% of introns containing most common branch point motif for given number of degenerate sites			
					0	1	2	3
*A. fumigatus*	All introns		18267	2.3	10.97% (actgac)	17.96% (ctgacw)	29.99% (ctracw)	43.18% (ctrayw)
	Orthologous	All	183	2.8 *	18.58% (actgac) *	28.42% (ctgacw)	41.53% (ctracw)	56.83% (ctrayw)
		Cons. to Ylip	171	2.8	19.30% (actgac)	28.07% (actrac)	40.94% (ctracw)	56.73% (ctrayw)
		Not Cons. to Ylip	12	3.6	33.33% (ctgaca)	50.00% (ctraca)	58.33% (ctraya)	75.00% (ctraym)
*S. pombe*	All introns		4722	3.9	21.61% (actaac)	35.26% (actaay)	50.46% (wctaay)	56.22% (wctray)
	Orthologous	All	125	3.7	22.40% (actaac)	33.60% (actaay)	53.60% (ctaayw)	61.60% (ctrayw)
		Cons. to Ylip	107	3.9	21.50% (actaac)	31.78% (actaay)	54.21% (ctaayw)	62.62% (ctrayw)
		Not Cons. to Ylip	18	3.3	27.78% (actaac)	44.44% (actaay)	50.00% (wctaay)	55.56% (wctray)
*A. thaliana*	All introns		134535	1.5	8.23% (tctgat)	14.95% (tctrat)	23.36% (wctrat)	30.99% (wctray)
	Orthologous		128	1.9	14.06% (ctgatt)	23.44% (ctratt)	30.47% (ctraty)	38.28% (ctrayw)
*H. sapiens*	All introns		197835	2.3	7.90% (ctgacc)	14.06% (ctgacy)	22.80% (ctgayy)	34.32% (ctrayw)
	Orthologous		157	3.2 *	8.70% (ctgact)	16.15% (ctgayt)	28.57% (tctray)	40.99% (wctray)
*T. gondii*	All introns		27085	2.5	4.54% (tctgac)	8.45% (tctgay)	14.06% (ctgayk)	17.75% (ctrayk)
	Orthologous	All	86	2.9 *	6.98% (ctgatg)	11.63% (ctgayg)	17.44% (ctrayg)	24.42% (ctrayk)
		Cons. to Cpar	61	2.8	4.92% (actaac)	9.84% (actaay)	14.75% (ctrayg)	21.31% (ctrayk)
		Not Cons. to Cpar	25	3.8	12.00% (tctgac)	24.00% (ctgayg)	28.00% (ctgayr)	32.00% (ctrays)

*: Indicates that the subset of orthologous introns is statistically significantly different at the P<0.05 level from all introns. For branch points, only 0-fold degeneracy was tested.

Despite this analysis being perhaps the most direct way to test the hypothesis of preferential intron retention available, it is deeply undermined by the great phylogenetic distances between intron-poor species and even their closest relatives (*T. gondii*-*C. parvum* and *A. fumigatus*-hemiascomycetes diverged both many hundred million years ago) and associated large amounts of sequence change. Therefore, the finding of a positive signal for any of these comparisons is surprising and intriguing. To further test whether the observed stronger intron consensus sequence signal in intron-poor organisms could truly reflect retained greater boundary strengths from ancestor, we further divided the orthologous set into those introns shared with a more closely related intron-poor species (*Y. lipolytica* for *A. fumigatus*; *C. parvum* for *T. gondii*) and those shared only with distantly related species. Whereas the former could conceivably retain some specific ancestral signal due to lack of change, the second set represent divergences dating back upwards of a billion years ago to early eukaryotic evolution, seemingly precluding similarities in trends across individual intron boundary strengths representing lack of sequence change since that time. Unexpectedly, we observed that this second subset of introns, shared only with older relatives, show stronger signal than those introns shared with the closest intron-poor ancestor in *T. gondii* (Table 4). This suggests that boundaries with greater strength in intron-poor species does not reflect retained ancestral signal. (There was an insufficient number of introns in the *A. fumigatus* distantly-related group for comparison).

Another argument also argues that these intron subsets' stronger boundaries reflects not retention of ancestral boundary strength, but something else: *A. fumigatus* and *Y. lipolytica* exhibit different 5′ splice site consensus sequences, thus while *A. fumigatus* introns with homology to retained *Y. lipolytica* introns do show greater homogeneity in 5′ splice site boundaries (matching the consensus GTAAGT), they do not more closely resemble *Y. lipolytica* boundaries (consensus GTGAGT). Indeed, we observe the opposite trend: only 15.2% (26/171) of the *A. fumigatus* introns shared with *Y. lipolytica* have a G in position +3, whereas in the whole set of *A. fumigatus* introns, 21.4% have a G in that position.

### PolyT Distributions

Finally, we studied the distribution of characteristic intronic polyT motifs along intron length. For each species, we calculated frequencies of intronic minimal “polyT motifs” (following previous studies, we define these as six consecutive nucleotides containing at least 3 T's and no A's [19],[21],[22]) as a function of distance from the acceptor site.

**Figure 6:**
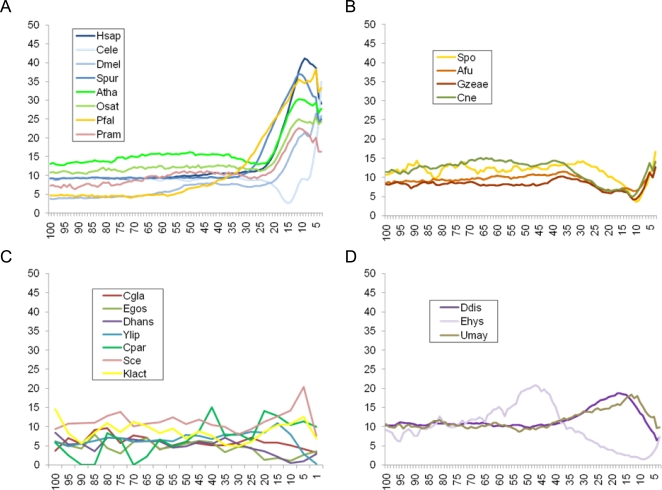
Characteristic patterns of polyT motif distributions across eukaryotes. Examples of: (A) Most common and widely distributed pattern. PolyT motifs concentrate at the 3′ of the introns. Observed in plants, animals, apicomplexa and heterokonts. (B) Pattern characteristic of most fungi and in *T. pseudonana*, characterized by relatively uniform polyT distribution. (C) Pattern of some intron-poor species. (D) Pattern observed in amebozoa and in the fungus *U. maydis*. Species included in the study: *Homo sapiens* (Hsap); *Caenorhabditis elegans* (Cele); *Drosophila melanogaster (*Dmel); *Strongylocentrotus purpuratus* (Spur); *Arabidopsis thaliana* (Atha); *Oryza sativa* (Osat); *Plasmodium falciparum* (Pfal); *Phytophthora ramorum* (Pram); *Schizosaccharomyces pombe* (Spo); *Aspergillus fumigatus* (Afu); *Giberella zeae* (Gzeae); *Cryptococcus neoformans* (Cne); *Candida glabrata* (Cgla); *Eremothecium gossypii* (Egos); *Debaryomyces hansenii* (Dhans); *Yarrowia lipolytica* (Ylip); *Cryptosporidium parvum* (Cpar); *Saccharomyces cerevisiae* (Sce); *Kluyveromyces lactis* (Klact); *Dictyostelium discoideum* (Ddis); *Entamoeba hystolitica* (Ehys); *Ustilago maydis* (Umay).

Almost all species conformed to one of 3 broad patterns, which tend to be conserved within large phylogenetic groups (Figure 6). For the most common distribution (found in metazoans, plants, most apicomplexa and the heterokont *Phytophtera* species) polyT motifs concentrate near the intron terminus (Figure 6A). The 5′ limit of the distribution is likely determined by branch point position in some species (∼30 nt, similar to mean BP-AG distance 31.5 nt in mammals [23] and 27.6 nt in *Arabidopsis thaliana* [24]). In other species (*Caenorhabditis elegans, Ciona intestinalis*, *Drosophila melanogaster,* and the apicomplexan *Theileria parva*) polyTs are concentrated in the last ∼10 nt, and are underrepresented 10–15 nt from the terminus. This pattern could suggest a more 3′ branch point position, although branch points in these species are difficult to determine [25]. The rhizarian *Plasmodiophora brassicae* seems consistent with this broad pattern; however, the small number of available introns renders confident conclusions difficult.

Second, in most fungi, polyTs are roughly equally common across the intron (with the exception of the position of the branch point site, which typically falls ∼13–25 nt from the terminus [19]) (Figure 6B). This pattern resembles that found in some intron-poor species, including the T-rich introns of *C. parvum* as well as the more moderate T-rich introns of hemiascomycete yeasts (Figure 6C). *S. cerevisiae* shows a partial exception to the pattern, with a pronounced peak ∼10 nt from the terminus.

The third pattern is found in the two fully-sequenced amoebozoans (*Dictyostelium discoideum* and *Entamoeba histolytica*) and in the intron-poor fungus *Ustilago maydis* (Figure 6D). This pattern shows a single peak in polyT occurrence, centered between 15 and 40 nt from the acceptor site.

Although the numerous exceptions make firm conclusions difficult, the broad phylogenetic distribution of the first pattern (in animals, plants, apicomplexans and heterokonts, and perhaps rhizarians), suggests that the ancestral intronic structure had polyT motifs concentrated between the BP and the terminal AG, and that broader polyT distributions evolved early in fungal evolution.

## Discussion

### Evolutionary Convergence to Highly-Conserved Intron-Exon Structures in Distant Eukaryotes

We report convergent evolution of strong branch point consensus sequences and constrained branch points positions in eukaryotic lineages ranging from fungi to plants to protists. These observations join our previous findings of convergent evolution to strong 5′ splice site boundaries [9], as well as the pattern of recurrent nearly-complete intron loss, as examples of convergent intron-exon structure evolution across eukaryotes [26]. Interestingly, these five patterns appear to be closely related. Those lineages that are highly derived in intron number, having lost most of their introns, are the same ones that exhibit constraint of their few remaining introns' sequences.

However, different intron sequence characteristics show different degrees of co-evolution with intron number. Whereas strong splice sites show a one-to-one correspondence with low intron number across species [9], only a subset of intron-poor lineages have strong branch point sequences, of which only a subset have highly constrained branch point positions. Thus while intron paucity may be necessary for the emergence of branch point sequence and position constraint, it is not sufficient.

Difference in levels of intron structure constraint likely is associated with (even perhaps driven by; see below) changes in the spliceosomal machinery that have led to increased requirements for adherence to consensus sequences [9],[27]. Indeed, the intron-poor species whose splicing apparatus has been most extensively studied, *S. cerevisiae*, shows considerable alterations in the mechanisms and protein components of its spliceosome [2],[19]. Future work should explore spliceosomal changes in other intron-poor lineages, in particular the possibility in evolutionary convergence in spliceosomal machinery across lineages.

Notably, we failed to find intermediate stages. 5′ splice site strength shows a clear gap between intron-poor lineages (at least 5 bits of information content), and intron-rich (1–4 bits) [9]. Almost all species have either clear and strong branch point consensus (66–100% introns with same branch point hexamer) or much weaker conservation (<30%). Branch point position also seems bimodal, as clearly seen among the hemiascomycetous yeasts, where either >80% of branch points fall within a few base pairs, or fewer than 40%.

For 5′ splice sites, this lack of intermediates is consistent with some qualitative difference in the selective regimes acting within intron-poor and intron-rich species, leading to a lack of intermediate strengths. For branch point sequences and positions the case is more subtle. Do weak branch points in some intron-poor lineages reflect an ongoing process, or are these lineages somehow refractory 3′ intron convergence? Repeated evolution of constraint in hemiascomycetes may suggest an ongoing process, however in this case we might expect to observe intermediate stages. Possibly, once put in motion, intron structure constraint proceeds rapidly, which could explain the lack of observed intermediates.

### Opposed Intron Structure Evolution in Two Classes of Reduced Eukaryotes

Widespread sequencing has underscored the complexity of eukaryotic genome structure. While some genomes seem generally complex (with large numbers of genes containing numerous long introns and ubiquitous transposable elements) or simple (with short intergenic regions flanking a modest complement of nearly intronless genes), many genomes defy such straight-forward characterization. Intron-exon structures provide a clear example: the three genomes with the shortest known intron structures each have relatively high intron densities (*Paramecium tetraurelia*, the nucleomorph of *B. natans*, and *Dicyemids*, so-called mesozoans), whereas introns in very intron-poor species are not particularly short (Table 2).

Interestingly, these two classes of reduced lineages appear to show opposed patterns of intron evolution. Whereas intron-poor lineages tend towards highly-constrained intron sequence elements, short-intron lineages seem to show very weak sequence constraint. Available *Dicyemid* introns give the weakest known score for 5′ intron boundaries (0.5 bits) and 5′ splice sites of *P. tetraurelia* and *B. natans* are largely restricted to GT(A). These three lineages also show no signature of branch points (Table 2). This does not simply reflect an inability of short introns to accommodate branch points or reduced splicing constraints associated with short introns per se: *E. cuniculi* introns (35.8 nt on average) and many *T. vaginalis* introns (∼25 nt) are short, yet both show conserved 5′ splice site and branch point sequences (note that this also suggests that species with both types of genome reduction exhibit strong consensus, reflecting their intron paucity). This pattern underscores the importance of intron number, and not simply genome reduction, in driving the emergence of strong consensus sequences.

### Hypotheses for Intron Convergence and a Natural Experiment in *Ostreococcus*

The finding of a general inverse correspondence between intron number and splicing signals' strength is unexpected and remains unexplained. Previously, we suggested that in intron-poor species, selection against aberrant splicing of cryptic splice sites would drive changes in the spliceosome towards stricter splicing requirements, which would in turn drive sequence change in (or loss of) non-conforming introns [9]. In intron-rich species, this evolutionary pathway would not be available since increased spliceosomal strictness would imply deleterious inefficient splicing of a much larger number of non-consensus introns [9].

The genome of the ultra-small green alga *Ostreococcus lucimarinus* provides a rare natural experiment to test this hypothesis. While genes in the majority of the genome exhibit very low intron densities, the genes spanning roughly half of chromosome 2 show a much higher density, well within that of “intron-rich” species [18]. That the two sets diverge so clearly in level of intron sequence constraint is clearly not predicted by general alterations of splicing strictness due to changes in a (assumed) single spliceosome.

One possibility is that the two intron sets are serviced by different spliceosomes (as is the case of U2 and U12 in different eukaryotic lineages). However, a computational search turned up only single copies of spliceosomal RNA components in congenitor *O. tauri* [28]. Conceivably snRNA changes to complement the divergent *Ostreococcus* 5′ splice sites and branch points (GTGCGTG and GACTGACG in *O. lucimarinus*) could have thwarted their identification in the previous study, and this possibility is worth exploring. Alternatively (though more difficult to test), a single core RNA splicing machinery could associate with different sets of protein components in distinct spliceosomes with different splicing activities.

More likely, however, *O. lucimarinus* contains a single spliceosome, strongly suggesting that the constrained introns through most of the genome, as well as those of intron-poor species that they so closely resemble, do not reflect inherent changes in the spliceosomal machinery. A simpler possibility is that differences in local (in *O. lucimarinus*) or cellular (in other species) concentrations of spliceosomal complexes is the driving factor. It seems possible or even likely that spliceosomal complexes in intron-poor species are downregulated. Such downregulation could reflect either selection to reduce incorrect splicing of truly exonic sequence (i.e. fewer spliceosomes, less chance of false splice boundaries being spliced), or could be favored by reducing energetic costs associated with transcription, processing, and translation of spliceosomal components. If so, the lowered concentration of spliceosomal components would require stronger binding affinity of individual splice sites to corresponding snRNAs for efficient splicing, which would in turn drive the evolution of stronger boundaries (or intron loss). Differential local concentrations across genomic regions in *Ostreococcus* could be maintained if spliceosomes were preferentially recruited to the intron-rich genomic region. This scenario is similar to our previous hypothesis in invoking a tradeoff between the costs of efficient splicing of weak boundaries (maintenance of high spliceosome concentration) and the costs of mis-splicing, which we argue would likely be different in intron-rich and -poor species. This hypothesis makes the testable prediction that spliceosomal components should show reduced expression in intron-poor species relative to intron-rich species.

Another hypothesis concerning the concentration of spliceosomal complexes, suggested to us by Tony Russell, sees a very different role for selection. A predicted consequence of increasing the length of snRNA-intron element base-pairing interactions is a reduction in the overall rate of splicing, simply due to a tighter association between intron and spliceosome. Such a decrease in splicing rate will be tolerated in intron-poor species if spliceosomal components are in excess relative to the number of introns. However, in intron-rich species spliceosomal components may not be in excess, in which case stronger base-pairing between intron and snRNAs could be disfavored. Notably, these two hypotheses make qualitatively different predictions. Whereas the latter hypothesis predicts that strong boundaries would be disfavored in intron-rich species, the former predicts that they would as or more fit than weaker boundaries. Comparative analysis of closely related species to test these predictions is underway.

### Sequence Convergence versus Preferential Loss

Two factors could drive evolutionary convergence to strong intron boundaries: sequence changes in existing intron sequences to consensus sequences, and preferential loss of non-consensus introns. The relative contributions of these factors may depend on the precise evolutionary pathway from (ancestral) genomes with many introns with weak boundaries and relatively lax splicing requirements to fewer introns with strong boundaries and stricter requirements.

First, widespread (mostly random) intron loss could lead to selective conditions favoring the evolution of a spliceosome with stricter sequence requirements for splicing (as argued above and in reference [9]). Introns with non-consensus sequences would then impose a burden, which could be resolved by sequence change or intron loss. If intron loss rates in these lineages are at least comparable to substitution mutation rates (for instance, 90% intron loss over 500 million years is consistent with a constant loss rate of 5×10^−9^ per year, comparable to some estimated mutation rates [29],[30]), preferential loss could play an (or even the) important role in convergence. In this case, intron loss would be a self-catalyzing process, with widespread intron loss leading to increased splicing requirements driving yet faster intron loss.

Alternatively, increased splicing requirements could come first, driving intron loss (consistent with [31]). However evolution of stricter splicing requirements in intron-rich organisms, where there are large numbers of non-consensus introns, would lead to widespread deleterious mis-splicing. Thus it is hard to imagine such strict requirements arising prior to widespread intron loss.

Finally, even under lax splicing requirements, loss of non-consensus introns could be more highly selected due to the effects of the less efficient splicing of these introns (e.g. [32]), with stricter splicing requirements gradually enabled by loss of non-consensus introns. The viability of this scenario depends on significantly higher selective costs of suboptimal boundaries in intron-rich species respect to the optimal introns. Attempts to estimate these selective costs by comparative analysis are underway.

In any case, it seems likely that intron loss, increased splicing constraints, and intron sequence change will all be reinforcing of one another, such that (perhaps past some critical threshold), the three will proceed in tandem. Differences in the relative contributions of the three phenomena will depend on mutation rates and selective coefficients for different kinds of changes (basepair substitutions, intron loss, spliceosomal changes), which may vary considerably across times and lineages.

### Convergent Evolution between U12-Type Introns and Nearly Intronless Species' U2-Type Introns

The convergent U2 intron structures observed here and elsewhere in some intron poor species – strong 5′ and branch point consensus sequences, constrained BP-AG length – are strikingly reminiscent of the U12-type intron structures found across a wide variety of lineages [33],[34]. One possibility is that the U12-type intron structures represent a derived state (possibly evolved in the ancestor of eukaryotes), and that these convergent cases have similar causes – that U12-type introns' low genomic number has subjected them to the same pressures as those experienced by U2-type introns in intron-poor lineages. Notably, if the conservation across lineages of specific conserved U12 intron sequence elements, as opposed to the differences in consensus structures observed in U2-type introns across diverse intron poor lineages reflects the emergence of these structures early on in eukaryotic history, this interpretation would imply that U12-type introns have been rare since very early in eukaryotic history. Alternatively, the similar structures of U12 introns in general and U2 introns in intron-poor species could have different explanations. One possibility is that the U12 system represents an intermediate between the type II introns that initially proliferated in early eukaryotic ancestors, with their highly similar sequences and structures, and typical highly degenerate U2 introns. In this case the persistence of strong consensus sequences in U12-type but not U2-type introns remains somewhat mysterious, as does the preponderance of U2 introns relative to U12.

### Evidence that polyT Tails and Weak Branch Point Sites Are Ancestral

We find different patterns of polyT motif distribution along the introns in different lineages, which likely reflect differences in polyT functionality and in how polyT-binding factors regulate splicing in these lineages. Indeed, the differential conservation and evolution of spliceosomal proteins binding polyT tracts (PTB, SXL, TIA1, Nam8, etc.) in each species is likely to determine the position of polyT motifs along introns and what role these motifs play in splicing regulation.

Among these locations, we find that the so-called “polyT tail”, spanning from a position likely corresponding to the branch point to the 3′ intron terminus, is common to a wide variety of groups ranging from plants to animals to various, widely diverged, protists, strongly suggesting that the existence of a polyT tail may be ancestral. Furthermore, we show that, as in the case of 5′ splice sites, strong branch point site consensus have evolved independently in only intron-poor species, whereas all intron-rich species have weak branch point sites. Since a wide variety of studies have shown that eukaryotic ancestors have harbored relatively high intron numbers [3], [35]–[38], our results suggest that the eukaryotic ancestors also had weak branch point site consensus, as with most modern eukaryotic groups.

These conclusions extend an excellent recent study from Schwartz *et al*. [27], who studied BP and polyY motifs in 19 opisthokonts and 3 other eukaryotic species, and reached similar conclusions about ancestral intron structures. Our inclusion of a more diverse set of species spanning all of known eukaryotic species allows us to reach deeper into eukaryotic evolution, potentially getting much closer to the initial origin of spliceosomal introns. In particular, we find that sequences from the first characterized rhizarian, as well as for various heterokonts, follow the patterns found in other kingdoms. This striking similarity over very broad evolutionary distances significantly strengthens our conclusions about ancestral eukaryotic splicing, rendering them independent of the placement of the root of the eukaryotic phylogeny.

The presence of these intronic features, polyT tail and weak branch point sites, in the eukaryotic ancestors adds to the developing picture of the spliceosomal system in early eukaryotes – with highly developed spliceosome, weak 5′ and branch point sequences, a polyT tail and complex splicing patterns [2],[9],[39],[40].

### Quality of Predictions

One limitation of the data deserves comment. We analyze annotated intron sequences, making our analysis subject to the quality of available annotations. Problems with the annotations may very directly influence the characteristics studied here, since for instance more consensus-like sequences are more likely to be identified as introns. Such concerns may also affect comparative analysis if the annotation efforts for different species are differentially sensitive to different kinds of introns.

However, it is very unlikely that such limitations are likely to drive the qualitative differences we see here. For a “weak” species with for instance 30% of its true introns exhibiting the same branch point to be incorrectly identified as a “strong” species (with, say, 80% of predicted introns with the same branch point) would require that the vast majority of its non-consensus introns have gone unannotated. For the reverse to occur, it would be required that there were so many falsely predicted introns that it had drowned out the signal almost entirely. Thus, while it is important to point out that our results are likely not accurate to the second decimal place due to problems with annotations, such problems are very unlikely to be driving the large qualitative differences observed.

### Strong 5′ Splice Sites in *E. cuniculi*

Possibly, the most important impact of annotation errors could be to reduce the signal in very intron-poor species. For instance, further scrutiny suggested that 2 of the 15 predicted introns in *E. cuniculi* may not in fact be introns at all: both are a multiple of 3 basepairs and lack inframe stop codons; these two introns have the weakest 5′ boundaries (matching the consensus (GT)AAGT at 1 and 2 out of 4 positions, compared to at least 3 matches for the other 13 introns); and one intron has similar sequences at the two boundaries, suggesting that this intron prediction could reflect a reverse transcriptase artifact in EST preparation [17]. Notably, in our previous work on donor splice sites [9], *E. cuniculi* represented the only intron-poor species lacking very clear strong boundaries. Excluding these two questionable introns, *E. cuniculi* has a donor site information content of 6.2 bits, comparable to the other intron-poor lineages.

### Conclusion

These results attest to plasticity of spliceosomal intron structures through the history of eukaryotes. The availability of large numbers of eukaryotic genomes now allows comparative analysis of an increasing diversity of genomic structures. Present and previous works have provided an increasingly detailed picture of the patterns and determinants of intron-exon structures, one of the hallmarks of eukaryotic genome organization. Definitive identification of the causes of highly regular 3′ intron structures awaits the identification of additional lineages exhibiting this pattern.

## Methods

### Genome Sequences and Data Sources

GenBank fully-sequence genome annotations were downloaded from NCBI webpage (http://www.ncbi.nlm.nih.gov) or Ensembl database (http://www.ensembl.org) for 7 metazoa: human (*Homo sapiens* (NCBI 36 Ensembl 38.36)), zebra fish (*Danio rerio* (Zv5 Ensembl 38.35e)), *Strongylocentrotus purpuratus* (AAGJ00000000.2), *Drosophila melanogaster* (release 4.1), mosquito (*Anopheles gambiae* (AgamP3 Vectorbase 38.3a)), *Caenorhabditis elegans* (WS150 Wormbase 38.150a), *Ciona intestinalis* (CINT1.95); 16 fungi: *Aspergillus fumigatus* Af293 (AAHF00000000.1), *Aspergillus nidulans* FGSC A4 (AACD00000000.1), *Neurospora crassa* OR74 A (AABX00000000.1), *Gibberella zeae* PH-1 (AACM00000000.1), *Yarrowia lipolytica* CLIB122 (CR382127-31.1), *Saccharomyces cerevisiae* YJM789 (AAFW00000000.1), *Candida glabrata* CBS138 (provided by J. E. Stajich), *Candida albicans* SC5314 (provided by J. E. Stajich), *Kluyveromyces lactis* NCYC 2644 (AADM00000000.1), *Eremothecium gossypii* ATCC 10895 (AE016814-20.1), *Debaryomyces hansenii* CBS767 (CR382133-9.1), *Schizosaccharomyces pombe* 972h (AL672256-8.1), *Ustilago maydis* 521 (AACP00000000.1), *Cryptococcus neoformans* B3501-A (NC_006670, NC_006679-87, NC006691-4), *Encephalitozoon cuniculi* GB-M1 (AL391737.1, AL590442-50.1), *Rhizopus oryzae* RA 99-880 (AACW00000000.2); 2 amoebae: *Dictyostelium discoideum* AX4 (AAFI00000000.1), *Entamoeba histolytica* HM-1:IMSS (AAFB00000000.1); 2 plants: *Arabidopsis thaliana* (NC_003070.5, NC_003071.3, NC_003074.4, NC_003075.3, NC_003076.4), *Oryza sativa* (Build 2.1); 3 green algae: *Chlamydomonas reinhardtii* (JGI Chlamy 3.0), *Ostreococcus lucimarinus* CCE9901 (GenBank version 1, CP000581- CP000601), Ostreococcus tauri OTH95 (GenBank version 1, CR954201- CR954220); 6 aplicomplexans: *Plasmodium falciparum* HB3 (AANS00000000.1), *Plasmodium yoelii yoelii* (AABL00000000.1), *Plasmodium chabaudi* (CAAJ00000000.1), *Theileria parva* Muguga (AAGK00000000.1), *Cryptosporidium parvum* Iowa (AAEE00000000.1) and *Toxoplasma gondii* RH (AAQM00000000.1); and 2 heterokonts: *Phytophthora ramorum* strain Pr102 (AAQX00000000.1) and *Phytophthora sojae* strain P6497 (AAQY00000000.1).

All reported intron sequences were obtained from published supplementary material for: *Guillardia theta NM* [41], *Cyanidioschyzon merolae* 10D [42], *Trichomonas vaginalis* [5],[16] and *Bigellowiella natans NM* [8] and *Giardia lamblia* ATCC 50803 strain WB C6 [43] (plus 2 introns we have identified), the mesozoan *Dicyema acuticephalum* [44], and the rhizarian *Plasmodiophora brassicae* [45].

Available gene sequences for unpublished or incomplete genomes were downloaded one by one as CDS annotations from NCBI web page (http://www.ncbi.nlm.nih.gov/): *Brassica oleracea* (1096 introns in 314 genes). Introns for *Paramecium tetraurelia* were extracted from nucleotide links of NCBI taxonomy (1082 introns in 401 genes). Introns for *Candida guilliermondii* (13 introns), *Candida lusitaniae* (10 introns) and *Candida tropicalis* (34 introns) were provided by J. E. Stajich.

### Study of Branch Point Consensus Strength

Studying the clear branch point consensus from a wide variety of intron-poor species, we first define an extended branch point consensus, WCTRAYN, consistent with the minimal consensus NYYNAN described for a wide variety of eukaryotic groups [19],[23],[24],[46].

For each 50 species, we next studied the percentage of introns showing the most common hexamer matching this extended consensus, allowing two-fold degeneracy at zero, one, two and three sites of the putative branch point hexamer. The four measures are complementary.

We did not aim to identify and study branch points in all introns. Instead, the percentage of use of the most common motifs gives a straight-forward measure of the strength of the signal for a given species. The use of a similar approach to measure the strength of the 5′ splice site (whose definition is trivial) shows a clear correspondence between the measure of strength as the percentage of introns with the most common sequence motif (i.e. GTAAGT, GTATGT, etc.) and as information content, used broadly in the literature, with a coefficient of correlation between both variables for the species included in this study is r = 0.96.

### Study of 5′ and 3′ Splice Site Consensus and BP-AG Distance

We aligned the final 20 nt of each intron for each species using WebLogo (http://weblogo.berkeley.edu/logo.cgi). To better characterize the evolution of BP-AG constraint in *Y. lipolytica*, we further studied BP-AG distance in the 9 hemiascomycetes species. In all hemiascomycetes species, the vast majority of introns contained a single TACTAAC sequence, used as the branch point. The BP-AG distance was defined as the number of base pairs (Nn) for 5′TACTAAC|Nn|AG-exon3′.

For 5′ splice sites we used a similar methodology as described in [9]. The first 6 bases of each intron were extracted, and information content in bits for positions +3 to +6 was calculated using PICTOGRAM software online (http://genes.mit.edu/pictogram.html).

### Study of *Ostreococcus* Introns

We downloaded available EST sequences for *O. lucimarinus* from NCBI on April 4^th^, and performed standalone BLASTN searches for each predicted intron-containing *O. lucimarinus* gene against the ESTs. Preliminary confirmed introns were identified as those in which an EST hit with >60 bits and with >90% sequence identity spanned the intron position (reaching at least 3 nt on each side of the intron position). Each of these introns was then analyzed by eye to exclude non-canonical intron positions as well as those for which sequence similarity between the regions spanning the 5′ and 3′ predicted splice boundaries were consistent with a template-switching artifact during the reverse transcription step of the EST library preparation [16],[17].

### Orthologous Intron Analysis

For each species considered, databases of intron/exon structures of predicted gene transcripts were prepared from the genome annotations. Homologs were identified by one-way BLASTP searches of intron-containing genes from intron-poor species against predicted proteomes from intron-rich species. Putatively orthologous introns were identified as those present at identical alignment positions (position and phase) in both species. Related species were defined as: *C. parvum* for *T. gondii*; *Y. lipolytica* (chosen as the representative hemiascomycete due to its greater intron density (3 times higher than *S. cerevisiae*), in order to increase sample size) for *S. pombe* and *A. fumigatus*; and the *C. merolae* and the *G. theta* nuclemorph for *A. thaliana.* Due to the lack of related intron-poor species, *H. sapiens* introns were divided into only two groups.

### Search for polyT Motif Distributions

We used the minimal definition for polyT motif, defined as six consecutive nucleotides containing at least 3 T's and no A's [19],[21],[22]. The study of introns for each species was performed using custom Perl scripts. The last 2 and the first 10 base pairs of each intron were excluded.

## References

[1] Vanacova S, Yan W, Carlton JM, Johnson PJ (2005). Spliceosomal introns in the deep-branching eukaryote Trichomonas vaginalis.. Proc Natl Acad Sci USA.

[2] Collins L, Penny D (2005). Complex spliceosomal organization ancestral to extant eukaryotes.. Mol Biol Evol.

[3] Slamovits CH, Keeling PJ (2006). A high density of ancient spliceosomal introns in oxymonad excavates.. BMC Evol Biol.

[4] Nixon J, Wang A, Morrison H, McArthur A, Sogin M (2002). A spliceosomal intron in Giardia lamblia.. Proc Natl Acad Sci USA.

[5] Carlton JM, Hirt RP, Silva JC, Delcher AL, Schatz M (2007). Draft Genome Sequence of the Sexually Transmitted Pathogen Trichomonas vaginalis.. Science.

[6] Roy SW (2006). Intron-rich ancestors.. Trends Genet.

[7] Jeffares DC, Mourier T, Penny D (2006). The biology of intron gain and loss.. Trends Genet.

[8] Gilson PR, Su V, Slamovits CH, Reith ME, Keeling PJ (2006). Complete nucleotide sequence of the chlorarachniophyte nucleomorph: Nature's smallest nucleus.. Proc Natl Acad Sci.

[9] Irimia M, Penny D, Roy SW (2007). Coevolution of genomic intron number and splice sites.. Trends Genet.

[10] Bon E, Casaregola S, Blandin G, Llorente B, Neuveglise C (2003). Molecular evolution of eukaryotic genomes: hemiascomycetous yeast spliceosomal introns.. Nucleic Acids Res.

[11] Blumenthal T, Steward K, Riddle D, Blumenthal T, Meyer B, Priess J (1997). C. elegans II;.

[12] Reed R, Maniatis T (1988). The role of the mammalian branchpoint sequence in pre-mRNA splicing.. Genes Dev.

[13] Query CC, Moore MJ, Sharp PA (1994). Branch nucleophile selection in pre-mRNA splicing: evidence for the bulged duplex model.. Genes Dev.

[14] Liang XH, Haritan A, Uliel S, Michaeli S (2003). trans and cis splicing in trypanosomatids: mechanism, factors, and regulation.. Eukaryot Cell.

[15] Hummel HS, Gillespie RD, Swindle J (2000). Mutational analysis of 3′ splice site selection during trans-splicing.. J Biol Chem.

[16] Roy SW, Irimia M (2008). When good transcripts go bad: artifactual RT-PCR ‘splicing’ and genome analysis.. BioEssays.

[17] Cocquet J, Chong A, Zhang G, Veitia RA (2006). Reverse transcriptase template switching and false alternative transcripts.. Genomics.

[18] Palenik B, Grimwood J, Aerts A, Rouze P, Salamov A (2007). The tiny eukaryote Ostreococcus provides genomic insights into the paradox of plankton speciation.. Proc Natl Acad Sci USA.

[19] Kupfer DM, Drabenstot SD, Buchanan KL, Lai H, Zhu H (2004). Introns and Splicing Elements of Five Diverse Fungi.. Eukaryotic Cell.

[20] Sheth N, Roca X, Hastings ML, Roeder T, Krainer AR (2006). Comprehensive splice-site analysis using comparative genomics.. Nucl Acids Res.

[21] Coolidge C, Seely R, Patton J (1997). Functional analysis of the polypyrimidine tract in pre-mRNA splicing.. Nucl Acids Res.

[22] Drabenstot SD, Kupfer DM, White JD, Dyer DW, Roe BA (2003). FELINES: a utility for extracting and examining EST-defined introns and exons.. Nucl Acids Res.

[23] Kol G, Lev-Maor G, Ast G (2005). Human-mouse comparative analysis reveals that branch-site plasticity contributes to splicing regulation.. Hum Mol Genet.

[24] Tolstrup N, Rouze P, Brunak S (1997). A branch point consensus from Arabidopsis found by non-circular analysis allows for better prediction of acceptor sites.. Nucl Acids Res.

[25] Zorio DAR, Blumenthal T (1999). Both subunits of U2AF recognize the 3′ splice site in Caenorhabditis elegans.. Nature.

[26] Roy SW, Gilbert W (2006). The evolution of spliceosomal introns: patterns, puzzles and progress.. Nat Rev Genet.

[27] Schwartz S, Silva J, Burstein D, Pupko T, Eyras E (2008). Large-scale comparative analysis of splicing signals and their corresponding splicing factors in eukaryotes.. Genome Res.

[28] Dávila López M, Rosenblad MA, Samuelsson T (2008). Computational screen for spliceosomal RNA genes aids in defining the phylogenetic distribution of major and minor spliceosomal components.. Nucl Acids Res.

[29] Tanabe K, Sakihama N, Hattori T, Ranford-Cartwright L, Goldman I (2004). Genetic distance in housekeeping genes between Plasmodium falciparum and Plasmodium reichenowi and within P. falciparum.. J Mol Evol.

[30] Neafsey DE, Hartl DL, Berriman M (2005). Evolution of noncoding and silent coding sites in the Plasmodium falciparum and Plasmodium reichenowi genomes.. Mol Biol Evol.

[31] Lynch M (2002). Intron evolution as a population-genetic process.. Proc Natl Acad Sci USA.

[32] Jaillon O, Bouhouche K, Gout J-F, Aury J-M, Noel B (2008). Translational control of intron splicing in eukaryotes.. Nature.

[33] Burge CB, Padgett RA, Sharp PA (1998). Evolutionary fates and origins of U12-type introns.. Mol Cell.

[34] Russell AG, Charette JM, Spencer DF, Gray MW (2006). An early evolutionary origin for the minor spliceosome.. Nature.

[35] Nguyen H, Yoshihama M, Kenmochi N (2005). New maximum likelihood estimators for eukaryotic intron evolution.. PLoS Comput Biol.

[36] Rogozin I, Sverdlov A, Babenko V, Koonin E (2005). Analysis of evolution of exon-intron structure of eukaryotic genes.. Brief Bioinform.

[37] Roy SW, Gilbert W (2005). Complex early genes.. Proc Natl Acad Sci USA.

[38] Sverdlov A, Rogozin I, Babenko V, Koonin E (2005). Conservation versus parallel gains in intron evolution.. Nucl Acids Res.

[39] Irimia M, Rukov JL, Penny D, Roy SW (2007). Functional and evolutionary analysis of alternatively spliced genes is consistent with an early eukaryotic origin of alternative splicing.. BMC Evol Biol.

[40] Roy S, Irimia M (2008). Intron mis-splicing: no alternative?. Genome Biology.

[41] Douglas S, Zauner S, Fraunholz M, Beaton M, Penny S (2001). The highly reduced genome of an enslaved algal nucleus.. Nature.

[42] Matsuzaki M, Misumi O, Shin-i T, Maruyama S, Takahara M (2004). Genome sequence of the ultrasmall unicellular red alga Cyanidioschyzon merolae 10D.. Nature.

[43] Morrison HG, McArthur AG, Gillin FD, Aley SB, Adam RD (2007). Genomic Minimalism in the Early Diverging Intestinal Parasite Giardia lamblia.. Science.

[44] Aruga J, Odaka YS, Kamiya A, Furuya H (2007). Dicyema Pax6 and Zic: tool-kit genes in a highly simplified bilaterian.. BMC Evol Biol.

[45] Bulman S, Ridgway HJ, Eady C, Conner AJ (2007). Intron-rich gene structure in the intracellular plant parasite Plasmodiophora brassicae.. Protist.

[46] Cavalcanti ARO, Stover NA, Orecchia L, Doak TG, Landweber LF (2004). Coding properties of Oxytricha trifallax ( Sterkiella histriomuscorum) macronuclear chromosomes: analysis of a pilot genome project.. Chromosoma.

[47] Simpson A, Roger A (2004). The real ‘kingdoms’ of eukaryotes.. Curr Biol.

[48] Keeling PJ, Burger G, Durnford DG, Lang BF, Lee RW (2005). The tree of eukaryotes.. Trends Ecol Evol.

